# Lipid Catabolism of Invertebrate Predator Indicates Widespread Wetland Ecosystem Degradation

**DOI:** 10.1371/journal.pone.0016029

**Published:** 2011-01-19

**Authors:** Michael J. Anteau, Alan D. Afton

**Affiliations:** 1 School of Renewable Natural Resources, Louisiana State University, Baton Rouge, Louisiana, United States of America; 2 Louisiana Cooperative Fish and Wildlife Research Unit, U.S. Geological Survey, Louisiana State University, Baton Rouge, Louisiana, United States of America; University of Queensland, Australia

## Abstract

Animals frequently undergo periods when they accumulate lipid reserves for subsequent energetically expensive activities, such as migration or breeding. During such periods, daily lipid-reserve dynamics (DLD) of sentinel species can quantify how landscape modifications affect function, health, and resilience of ecosystems. *Aythya affinis* (Eyton 1838; lesser scaup; diving duck) are macroinvertebrate predators; they migrate through an agriculturally dominated landscape in spring where they select wetlands with the greatest food density to refuel and accumulate lipid reserves for subsequent reproduction. We index DLD by measuring plasma-lipid metabolites of female scaup (n = 459) that were refueling at 75 spring migration stopover areas distributed across the upper Midwest, USA. We also indexed DLD for females (n = 44) refueling on a riverine site (Pool 19) south of our upper Midwest study area. We found that mean DLD estimates were significantly (P<0.05) less than zero in all ecophysiographic regions of the upper Midwest, and the greatest negative value was in the Iowa Prairie Pothole region (-31.6). Mean DLD was 16.8 at Pool 19 and was markedly greater than in any region of the upper Midwest. Our results indicate that females catabolized rather than stored lipid reserves throughout the upper Midwest. Moreover, levels of lipid catabolism are alarming, because scaup use the best quality wetlands available within a given stopover area. Accordingly, these results provide evidence of wetland ecosystem degradation across this large agricultural landscape and document affects that are carried-up through several trophic levels. Interestingly, storing of lipids by scaup at Pool 19 likely reflects similar ecosystem perturbations as observed in the upper Midwest because wetland drainage and agricultural runoff nutrifies the riverine habitat that scaup use at Pool 19. Finally, our results underscore how using this novel technique to monitor DLD, of a carefully selected sentinel species, can index ecosystem health at a landscape scale.

## Introduction

### Prairie Wetlands

Large proportions of wetlands in Iowa, Minnesota, and North Dakota have been drained or otherwise lost in the past 200 years [1]. In this modified agricultural landscape, remaining wetlands typically are large, permanent, and subject to various perturbations (e.g., ditches, drainage tile, sedimentation, cultivation) that degrade wetland quality [2]. The degree of wetland drainage or perturbation varies throughout the upper Midwest; proportionally more wetlands have been drained, and the surrounding upland landscapes are more intensely farmed in Iowa and southern Minnesota than in North Dakota [1], [3].

Prairie wetlands exist in a network of hydrologically connected basins and the removal or perturbation of small-less-permanent wetlands influences ecosystem functions of large-more-permanent wetlands [4]. Large permanent or semipermanent wetlands may exist in either a clear-water state, dominated by submerged-aquatic vegetation and abundant macroinvertebrates, or in a turbid state, dominated by phytoplankton and with depressed populations of macroinvertebrates [5], [6]. Large wetlands frequently are turbid in disturbed landscapes, where wetlands are prone to invasion of non-native or invasive species and receive inputs of sediments and nutrients from agricultural activities [7], [8]. Clear water wetlands generally are considered greater quality in terms of their ecosystem function [3], [7]. Factors influencing trophic structure and wetland quality have important implications for ecosystem services, conservation, and management of wetlands and surrounding upland landscapes in the upper Midwest, USA [3], [7]. The extent that drainage and perturbation influences wetland ecosystem processes or functions has not been evaluated within the upper Midwest and represents a critical research need. We predict that the quality of current ecosystem processes or functions would vary among regions of the upper Midwest and be related to relative levels of perturbations.

### Riverine Wetlands

Concurrent with development of industrialized agriculture in the upper Midwest, nutrient levels in the Mississippi River have increased markedly [9]. Agricultural fields in the upper Midwest have little vegetative cover to stabilize soil and nutrients in spring, a period when surface water runoff from snow-melt and precipitation runoff are relatively great. Drainage of wetlands in the upper Midwest decreases upper catchment storage and further facilitates transport of nutrients from agricultural fields into riverine wetlands [9], [10]. Accordingly, prairie and riverine wetlands experience increased inputs of sediment or nutrients in response to increases in the intensity of agricultural landscape changes.

### The Macroinvertebrate Predator and its Prey

Animals frequently undergo periods in their life cycle when they accumulate lipid reserves to be used for subsequent energetically expensive activities, such as migration or breeding. We propose that daily lipid-reserve dynamics (DLD) of sentinel species, during periods that necessitate lipid accumulation, can quantify how landscape modifications affect function, health, and resilience of ecosystems.

*Aythya affinis* (Eyton 1838; lesser scaup; hereafter scaup) are an ideal sentinel species for prairie and riverine wetland ecosystems for several reasons. Up to 86% of the scaup population migrates through the prairies of the upper Midwest in spring [11], [12] and use riverine and semipermanent and permanent wetlands of the prairies to replenish nutrient reserves after migratory flights (refuel) and to accumulate lipid reserves subsequently used for reproduction in more northern latitudes [13], [14], [15]. Scaup specialize on consuming aquatic macroinvertebrates, and amphipods (*Gammarus lacustris* and *Hyalella azteca*) are their preferred and historically predominant food in prairie wetlands during spring and early summer [16], [17], [18], [19]. However, mollusks can be a profitable food when they are very abundant. Unlike many other waterfowl species, scaup do not forage in terrestrial environments and generally do not switch to alternative food resources, such as seeds and fish that potentially are available in turbid wetlands [18]. In spring, scaup migrate relatively slowly through the upper Midwest [20], allowing them to sample and select wetlands with greater densities of amphipods [19]. Additionally, amphipods are important indicators of water and wetland quality, given their sensitivity to contaminants, pesticides, pollution, sedimentation, and non-native-invasive species [7], [21], [22].

Although historical data on amphipod populations are sparse, their populations seemingly have declined in the upper Midwest, which would indicate a degradation of wetland ecosystem processes in lower trophic levels [7]. An important question is whether or not amphipods and other macroinvertebrates in the upper Midwest have indeed declined to the extent necessary to negatively affect macroinvertebrate predators feeding in higher trophic levels. Catabolism of lipid reserves, rather than accumulation, by scaup on stopovers across the upper Midwest would 1) be consistent with previous evidence of a decline in amphipods and other macroinvertebrates, 2) provide evidence of aquatic ecosystem degradation throughout lower trophic levels in this landscape, and 3) indicate that effects of this degradation are evident in higher trophic levels in the ecosystem.

Pool 19 of the Mississippi River is an important riverine wetland stopover area [20], [23] where scaup traditionally consumed abundant bivalve prey [16]. We suspect that bivalve abundance has increased at Pool 19 due to nutrient enrichment of the Mississippi River. Further, we predicted scaup would store lipids at Pool 19 because they can forage profitably upon abundant bivalve prey. Increases in bivalve densities at Pool 19 should improve lipid accumulation in scaup, but ironically would be further suggestive of ecosystem degredation in the prairies of the upper Midwest.

Plasma-lipid metabolites (triglyceride and β-hydroxybutyrate; hereafter TRIG and BOHB, respectively) predict accumulation or catabolism of lipids in birds [24] and have been used to evaluate habitat quality of avian species [25], [26], [27]. Specifically, plasma-lipid metabolites can predict daily lipid dynamics (DLD) in free living scaup on spring migration stopover areas [28].

**Figure 1:**
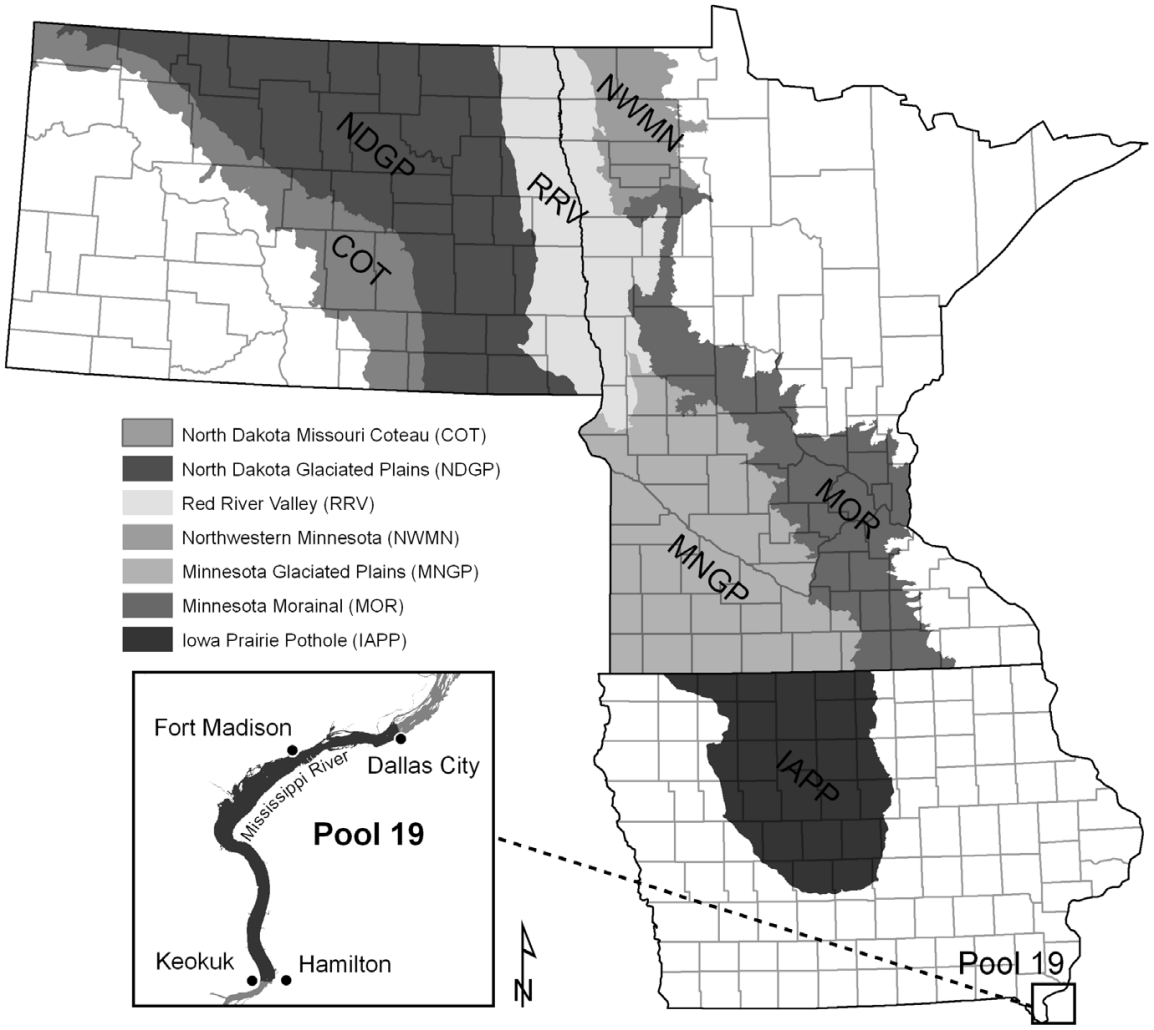
Study area depicting 7 eco-physiographic regions in the upper Midwest. Areas within these eco-physiographic regions were selected randomly for collections of lesser scaup blood samples during springs 2004–2005. Areas in white were not sampled.

Here we measured DLD of lesser scaup to determine where lipids were accumulated or catabolized during migration stopovers throughout the upper Midwest (Fig. 1) with the goal of indexing wetland ecosystems functions. We also contrasted DLD indexes of scaup from the upper Midwest to that from Pool 19 of the Mississippi River.

## Methods

We randomly selected collection sites annually within our stratified study area (Fig. 1) [20]. Each collection site (27,972 ha) was comprised of 3 townships (9,324 ha) that were within 50 km the first selected township. In 2004–2005, we collected 6–10 female lesser scaup from each site and 25 females annually at Pool 19; previous studies indicated that these sample sizes would be adequate to detect important differences in body-condition dynamics of scaup [13], [15], [20].

We collected 459 females randomly with a shotgun across the upper Midwest during the middle of spring migration for scaup; we never used decoys because they can bias body condition results [20], [29]. At Pool 19, we collected 19 and 25 females in springs (March 21–27) 2004 and 2005, respectively. In the upper Midwest, we collected females that were observed foraging, avoiding morning collections (2–3 hours after sunrise), which ensured that birds collected were not influenced by nocturnal fasting or recent migratory flights [30], [31]. At Pool 19 we collected female scaup randomly at night (11:00 pm–3:00am) from large flocks using a boat and spotlight. Nocturnal collections of female scaup ensured a random sample and eliminated daytime disturbances to people living along Pool 19 [13]. DLD of birds collected at Pool 19 might be influenced by nocturnal fasting or long migratory flights [30], [31], thus our estimates of DLD at Pool 19 could be biased toward smaller values.

Immediately after collection, we extracted 1.5 ml of blood from specimens by cardiac puncture using a 3.8-cm-20-gauge needle. We handled, centrifuged, and stored blood samples using established protocols [28]. We excluded 20 plasma samples where hemolysis occurred [32]. We conducted assays of TRIG and BOHB [33] on samples of 439 females from the upper Midwest (Table 1) and 44 females from Pool 19. For each individual, we indexed DLD from TRIG and BOHB with a predictive equation for scaup [28].

**Table 1:** Regions, years, dates, and numbers of female lesser scaup (N) collected during spring migration in the upper Midwest, numbers of collection sites (S), and townships where collections occurred (T).

Region	Year	Dates	N	S	T
Iowa Prairie Pothole	2004	6–9 April	26	3	3
	2005	30 March–4 April	22	3	6
Minnesota Morainal	2004	10–15 April	12	4	4
	2005	8–16 April	30	4	5
Minnesota Glaciated Plains	2004	8–19 April	30	4	7
	2005	10–15 April	33	4	7
Northwestern Minnesota *	2004	4–13 May	44	3	3
	2005	23–28 April	33	3	3
Red River Valley	2004	24 April–2 May	16	3	4
	2005	17–21 April	25	3	3
North Dakota Glaciated Plains	2004	20 April–11 May	44	6	9
	2005	9 April–2 May	63	6	11
North Dakota Missouri Coteau	2004	15–22 April	27	3	5
	2005	7 April–1 May	34	3	5

*Fixed collection sites (same each year).

TRIG concentrations (mmol*L^−1^) for females collected at Pool 19 and in the upper Midwest ranged from 0.196–6.927, and BOHB concentrations (mmol*L^−1^) ranged from 0.151–3.849. Ninety-three and 98% of TRIG and BOHB concentrations, respectively, were within the range of those used to establish the model for predicting DLD [28]. Accordingly, applying the predictive model to our data would produce reliable estimates of DLD.

We tested for regional variation in DLD using analysis of variance (PROC MIXED) [34], including year and sub-region within region as class variables. We used t-tests within the LSMEANS statement to determine if regional estimates of DLD differed from zero (*alpha*  = 0.05) [34]. We also calculated Tukey-Kramer adjusted least-square means and letter groupings for each region and year (*alpha*  = 0.05) [34].

## Results

**Figure 2:**
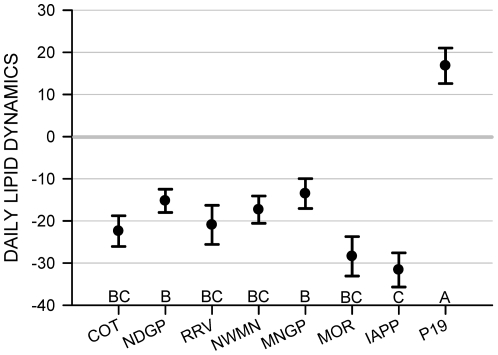
Mean daily lipid dynamics (DLD ± SE) of female lesser scaup in the upper Midwest. Least-square mean daily lipid dynamics (DLD ± SE) of female lesser scaup (n = 483) collected from stopover sites during spring migration 2004 and 2005 by regions (COT  =  ND Missouri Coteau, NDGP  =  ND Glaciated Plains, RRV  =  Red River Valley of MN and ND, NWMN  =  Northwestern MN, MNGP  =  MN Glaciated Plains, MOR  =  MN Morainal, IA  =  IA Prairie Pothole, and P19  =  Pool 19). Capital letters are Tukey-Kramer adjusted mean grouping at *P*<0.05. DLD values >0 indicate lipid storage while those <0 indicate lipid catabolism.

Mean DLD varied among regions (F _7, 458_ = 12.56, *P*<0.001), among sub-regions within regions (F _16, 458_ = 2.59, *P*<0.001), and between years (F _1, 458_ = 12.83, *P*<0.001). Mean DLD estimates differed from zero in all regions (all *P*s<0.05) and were less than zero, indicating catabolism, in all regions of Iowa, Minnesota, and North Dakota (Fig. 2). However, mean DLD was 16.8 at Pool 19 and markedly greater than in any other region (Fig. 2). Mean DLD in the Iowa Prairie Pothole region (−31.6) was less than it was in North Dakota Glaciated Plains and Minnesota Glaciated Plains (−15.2 and −13.5, respectively; Fig. 2). Overall mean DLD during 2004 (−21.3, SE  = 1.99) was less than it was during 2005 (−11.8 g, SE  = 1.73).

## Discussion

### Lipid-Reserve Dynamics in the upper Midwest

Prior to the 1980s, female scaup accumulated or at least maintained lipid reserves while migrating across the upper Midwest [13]. Our results are consistent with those published earlier [20], which indicated that female scaup currently have markedly fewer lipid reserves throughout the upper Midwest than do those at Pool 19. However, body mass and lipid, protein, and mineral reserves, examined in previous studies, are influenced by a combination of environmental factors at specific collection sites and at preceding migration stopover areas [13]. Moreover, nutrient-reserve levels could be influenced by the length and duration of migration flight prior to the arrival on a staging area [20]. In contrast, plasma-lipid metabolites provide a real-time assessment of bird condition and are more appropriate for making inferences about habitat quality where the birds were sampled [24]. Until recently, interpreting plasma-lipid metabolite data has been subjective, but now we can discriminate lipid accumulation and catabolism and make comparisons among varying degrees of catabolism or accumulation [28].

The observed widespread catabolism of lipid reserves by females likely is caused by a decrease in the availability of energy-dense food resources for scaup (e.g., amphipods) on spring stopover areas in the upper Midwest. Regions of the upper Midwest where females had the greatest rate of lipid catabolism generally were the same regions that had the lowest densities of amphipods [7]. Concurrent with the apparent decline in amphipod densities across the upper Midwest [7], [35], percentages of amphipods in current scaup diets throughout Minnesota and Iowa were less than those reported historically, and scaup consumed, on average, 49–52% less food (dry mass) throughout the upper Midwest than they did historically [17], [18]. Accordingly, amphipods and wetland quality apparently have declined to a level that has affected species that feed at higher trophic levels in prairie wetlands.

Amphipod and other macroinvertebrate densities are relatively greater in less turbid and altered wetlands; specifically, they are positively correlated with submerged aquatic vegetation [33], [36] and negatively correlated with both fish densities [33], [37] and elevated levels of suspended sediments[33]. Thus, lipid catabolism in our sample of scaup is consistent with substantial degradation of wetlands ecosystem processes and related declines in amphipod densities throughout the upper Midwest [7]. Moreover, the observed levels of lipid catabolism in scaup are alarming, given that scaup selectively use wetlands with the greatest densities of amphipods within the landscape, and thus, our data likely represent the greatest quality wetlands available within a given stopover area [19].

### Connections among Wetland Basins

Macroinvertebrate densities vary inter-annually in a given wetland, in response to varying water regimes and winter severity. Wetland productivity typically peaks in years following a return of wet conditions and declines through years of high and stable water levels [38]. Overall densities of *Hyalella azteca* (amphipods) in semipermanent and permanent wetlands across the upper Midwest were greater in 2005 than those in 2004 [7], which may explain why scaup catabolized fewer lipid reserves in 2005 than in 2004. Lipid catabolism rates that we documented could have been related to natural decreases in invertebrate abundance due to a reduced productivity phase in the wet-dry cycle. However, our upper Midwest study area is large enough that it encompasses areas with differing climate regimes [39], whereas, we observed lipid catabolism across the entire upper Midwest landscape. Natural variation from wet-dry cycles may influence lipid-reserve dynamics of scaup, but does not explain the observed consistent pattern of lipid catabolism or consistently low amphipod densities [7]. Further, a long term examination of wetlands in prairie Canada suggested that wetland degradation had occurred there throughout inter-annual fluctuations in water levels [40]. Consolidation drainage is a common practice of draining smaller less permanent wetlands into larger more permanent ones [41]; it decreases upper-catchment storage and increases connections among wetlands, likely producing high and stable water regimes in consolidated basins. Therefore, it is important to understand if landscape modifications have attenuated water-level fluctuations that historically occurred during wet-dry cycles.

### Connections with the Mississippi River

Large numbers of scaup stop at Pool 19 during spring migration and stay there for up to 4 weeks [23]. Our results were consistent with predictions that females at Pool 19 accumulate lipid reserves, and prior observations of females there having relatively large lipid reserves [13], [20]. At Pool 19, scaup forage on bivalves which are relatively less nutritious than are amphipods [17]; however, bivalve densities apparently were great enough to be nutritionally and energetically profitable. Moreover, our collections at Pool 19 were at night and females may have been fasting because few had diet samples in their upper-digestive tract (15% of females collected 2000–2005; M. J. Anteau, unpublished data). Thus, our estimate of lipid accumulation at Pool 19 is conservative [42].

We suspect that landscape modifications in the upper Midwest have increased food availability for scaup at Pool 19. Nutrient levels of water flowing in the Mississippi River have increased markedly since the 1930s and now they peak in spring; whereas, they historically were stable throughout the annual cycle [9]. Bivalve abundance at Pool 19 is positively correlated with the flows of the Mississippi River during the previous spring [43], suggesting that greater spring runoff over agricultural fields in the upper Midwest carries more nutrients important for bivalve production at Pool 19 [10], [44]. Accordingly, our index of DLD likely reflect the same perturbations (i.e., wetland drainage and agricultural runoff) that drive differing ecological processes in these two systems; ultimately, indicative of reduced wetland quality in the upper Midwest.

### Evaluating Ecosystem Function

Evaluating DLD patterns of scaup provides information about general habitat quality and ecosystem function across a large landscape and can provide a basis for prioritizing conservation or restoration activities. Our results suggest that wetland ecosystems are most degraded in Iowa and the Morainal region of Minnesota, which is consistent with the relative intensity with which that landscape has been altered through agricultural processes and invasive fish species [3], [7].

Ecosystem services of wetlands in the upper Midwest have become increasingly important, including: water quality improvement, flood control, ground water recharge, biodiversity, nutrient cycling, carbon sequestration, reduction of sediment and nutrient loading in rivers, and recreational opportunities [3], [45], [46]. Additionally, wetlands in the upper Midwest provide habitat for breeding migratory birds and other resident wildlife, and have the potential to influence populations of migratory birds that breed throughout North America because wetlands in the upper Midwest are important stopover habitats during spring and fall migration. Lipid-reserve accumulation by scaup and amphipod densities apparently are linked with many, if not all, of the ecosystem services provided by wetlands. For example, conservation activities to stop loss and degradation of wetland ecosystems, including a focus on improving amphipod abundances throughout the upper Midwest should improve lipid-reserve accumulation of female scaup and may help reverse this species' current continental population decline [12], but also would be beneficial to other ecosystem services important to humans. Moreover, there is a strong need to understand how ecosystem services of wetlands throughout the upper Midwest are influenced by the apparent ecosystem degradation signal in our data.
